# e-Learning for enhancement of medical student performance at the Objective Structured Clinical Examination (OSCE)

**DOI:** 10.1371/journal.pone.0253860

**Published:** 2021-07-01

**Authors:** Kyong-Jee Kim, Yeon Ji Lee, Mi Jin Lee, Young Hyo Kim

**Affiliations:** 1 Department of Medical Education, Dongguk University School of Medicine, Goyang, Republic of Korea; 2 Department of Family Medicine, Inha University School of Medicine, Incheon, Republic of Korea; 3 Department of Medical Education Center, Inha University School of Medicine, Incheon, Republic of Korea; 4 Department of Education, Graduate School, Inha University, Incheon, Republic of Korea; 5 Department of Otorhinolaryngology-Head and Neck Surgery, Inha University School of Medicine, Incheon, Republic of Korea; University of Botswana Faculty of Medicine, BOTSWANA

## Abstract

This study aimed to investigate the impact of student e-learning on the development of clinical competencies. The study participants were 3^rd^ year students (*n* = 43) at a private mid-sized medical school located in a South Korean suburb on a four-year medical program. Educational intervention was implemented to enhance student clinical performance. Students engaged in learning activities that intended to promote their self-directed learning abilities and clinical performances using e-learning resources. Intervention was conducted for the duration of six months during the 3^rd^ year and its effectiveness was investigated by comparing student performances in OSCEs in a pre- and post- comparison format and also by comparing them with national scores. In addition, student perceptions of the impact of e-learning on their OSCE performances were assessed using a questionnaire, which included 36 items that elicited student perceptions of their experiences of e-learning and readiness for e-learning. Student OSCE scores improved significantly after educational intervention in all domains of clinical competencies assessed and for total scores (*p* < 0.001). Furthermore, students achieved higher OSCE scores than national average scores in the post-test, whereas they had performed lower than national average scores in the pre-test. Students showed neutral or slightly positive responses to the effectiveness of e-learning, and their perceptions of e-learning were not associated with their e-learning readiness scores. The study shows student OSCE performance improved significantly after educational intervention, which indicate the effectiveness of e-learning to support student learning of clinical performance. Despite significant improvements in student OSCE scores after e-learning, their perceptions of its effectiveness were neutral. Furthermore, student perceptions of e-learning were not associated with their readiness for it. Suggestions are made to help students use e-learning more effectively to enhance their clinical competencies.

## Introduction

Clinical education is increasingly facing challenges that call for innovation. Given the COVID-19 pandemic, there is an urgent need for innovation in the teaching and learning of medicine [1]. In particular, medical schools are obliged to respond to the need for clinical education among students with limited opportunities for first-hand patient experiences while clinical clerkships are suspended due to the pandemic. As such, online learning has attained a position of prominence in medical education [2]. Second, there is a mismatch between medical students’ learning needs and the realities of today’s clinical settings [3, 4]. Clinical education at tertiary academic medical centers is not suitable for teaching medical students the competencies required of primary physicians at the basic medical education phase [5]. Moreover, clinical experiences involving engagement in clinical encounters ranging from patient presentation to making diagnosis and treatment plans are limited for medical students during clerkships [6, 7], and such problems are likely to be exacerbated during the COVID-19 pandemic.

Although the Objective Structured Clinical Examination (OSCE) is an important to tool for assessing student clinical competencies, students frequently lack opportunities to practice, other than in the high-stakes OSCE examination [8]. Furthermore, some medical schools have moved the teaching and assessments of clinical skills into online formats, including virtual OSCEs [9, 10], in response to the COVID-19 pandemic. Under these circumstances, it is likely that there will be fewer opportunities to teach and learn clinical performance, and thus, to prepare students for clinical performance tests. Therefore, there is a need for educational interventions that supplement teaching and learning, promote student clinical competencies, and help them prepare for OSCEs.

The literature suggests that reflection and self-directed learning are of paramount importance for effective learning [11] and are key to promoting the development of professional competencies [12]. Likewise, previous studies indicate that reflection and self-directed learning improve student OSCE scores [13, 14]. e-Learning is becoming increasingly important in medical education and is an integral part of supporting medical students’ self-directed learning as in MOOCs (Massive Open Online Courses) [15] and other online learning resources [16–18]. Still, research is scant on the use of e-learning for self-directed learning to promote student clinical competencies and its impact on student performance in OSCEs. Thus, this study aimed to investigate the impact of e-learning on student self-directed learning and the development of clinical competencies.

e-Learning, which is often used interchangeably with online learning or distance learning [19], is known to be at least as effective as or even better than traditional instruction in medical education [20, 21]. However, research on e-learning in the clinical education setting is scant, although the effectiveness of online videos for the learning of clinical skills has been well demonstrated [22, 23]. Proliferation of the use of technology due to the COVID-19 pandemic has generated an increasing need for research on the effective use of e-learning in clinical education. Cook [24] asserts that research on e-learning needs to focus on when and how to use it effectively rather than on head-to-head comparisons of its effectiveness with traditional instruction.

Research indicates that individual factors need to be taken into account when designing and implementing effective online learning environments [25]. One of the factors that influence effective e-learning is learner readiness. Several instruments have been developed to measure college student readiness for online learning [26]. Hung and colleagues [27] developed and validated an instrument for measuring learner readiness for online learning that consisted of five domains. They found college student levels of online learning readiness were high for computer/internet and online communication self-efficacy, and motivation for learning, but were low for learner control and self-directed learning [27]. Still, little research has been performed on medical students’ readiness for e-learning or on its impacts on their perceived effectiveness of e-learning.

In this study, the research questions asked were:

- Does student OSCE performance improve after they engage in e-learning?
- Does student readiness for e-learning affect their perceptions of its effectiveness?

## Materials and methods

### Study participants and the study setting

An ethical review was conducted and approved by the institutional review board of Inha University School of Medicine (IRB approval number: 2020-06-036). Informed consent was obtained from the participants. Study participants were 3^rd^ year students (*n* = 43) at a private mid-sized medical school located in a suburb in South Korea. The school has a four-year basic medical education curriculum, and the 3^rd^ year curriculum ran from January 2019 till February 2020, during which students attended core clerkships. Students underwent OSCEs twice during the Year 3 curriculum. The OSCE comprised six stations with 10 minutes allocated for each station at which they interacted with Standardized Patients (SPs). Students were assessed for their clinical competences in history-taking, physical examinations, and patient-doctor relationships (patient-physician interactions), which were assessed by SPs.

We designed and implemented an educational intervention implemented for Year 3 students in 2019 to promote the development of students’ clinical competencies as their performance in the first round of OSCE was short of the national average. This e-learning course ran in a self-directed learning format for the duration of six months and was awarded one credit-hour. Previously, the school did not offer any formal course in clinical performance during the Year 3 program, except for the learning during clinical rotations. In this new course, students engaged in online learning activities intended to promote their reflection and learning of clinical performances. A face-to-face orientation session was offered for the students from the course director for the duration of one hour before the e-learning course began. The instructor fully explained the purpose of this study to the students and introduced them to the e-learning website. The instructor also explained the parts of the video clips that should be watched with special attention to improve student performance.

Students engaged in two learning activities in this e-learning course. First, students wrote a reflective paper on their OSCE performance to promote their self-assessment of his/her clinical performance. For this reflection students first reviewed their clinical performances by watching video clips of themselves at OSCE stations and then rated their clinical performances using a 5-point Likert-type scale in 14 areas from history-taking, physical examinations, to patient-doctor relationships, and overall performance. Based on this self-assessment, students wrote a reflective paper on their OSCE performances. Second, students engaged in learning of clinical performances using e-learning resources. The e-learning resources were provided by the Korean Consortium for e-Learning in Medical Education (www.mededu.or.kr), which were developed and shared by nationwide medical schools. This website offers online clinical videos with duration approximately 10 minutes per clip that show patient encounters for over 40 clinical presentation topics and demonstrate the entire process of patient encounters in a clinic setting. Further information on these video resources is provided elsewhere [18]. After watching online videos, students analyzed the patient encounter presented in the video clip, excluding each differential diagnosis through medical history and physical examination of the SP, and created a schematic leading to the final diagnosis. Students wrote a report of their analysis of patient encounters from at least six video clips and were free to choose any subject from a repository of over 40 clinical presentations based on their individual learning needs.

### Study procedures

Students took the OSCE twice during the Year 3 curriculum. The first OSCE took place in June 2019 approximately five months after they had begun attending clinical rotations. The second OSCE was taken in February 2020 when the Year 3 curriculum ended. Between the two OSCE tests, students participated in the e-learning course while also attending clinical rotations. Students took the e-learning course on their own schedule in the self-directed learning format.

To investigate the effectiveness of e-learning intervention, student performance at OSCE tests in Year 3 were assessed using a pre-post comparative format. Student OSCE scores were also compared with national scores to investigate improvements in performances. Student perceptions of the impact of e-learning on their OSCE performances were assessed using a questionnaire. For this purpose, we developed a questionnaire that included 36 items composed of three sections. The first section addressed respondent demographic information, and the second section consisted of 10 items that elicited participants’ perceptions of their experiences of e-learning. The third section contained 12 items (adapted from Hung [27]) that assessed student readiness for e-learning, which comprised of three sub-scales that addressed (a) self-directed learning, (b) learner control, and (c) motivation for learning. We translated the original questionnaire, which was written in English into Korean, as we had experience in medical education research and Korean translation. The translation was reviewed by experienced medical educators to check for the clarity of items.

Students responded to the statements in the second and third sections of the questionnaire using a five-point scale, where 1 = “Strongly Disagree,” and 5 = “Strongly Agree.” The questionnaire was self-administered and implemented using an online survey tool in June 2020. The implementation of the online survey was rescheduled from the beginning to the end of the semester in spring 2020 due to the urgent needs for responding to the curricular changes caused by the COVID-19.

### Data collection and ethical considerations

Student OSCE scores in the domains of history-taking, physical exams, and patient-doctor relationships were obtained for the first and second tests and were summed to produce total scores with a maximum possible score of 100. Student OSCE scores were compared with those of students at other medical schools who had taken OSCEs administered by a consortium of 18 medical schools in the Seoul metropolitan area.

Questionnaires were administered after obtaining permission from the Institutional Review Board of Inha University School of Medicine (2020-06-036). We provided all participating students with a description of the purpose and methods of the study and stressed their rights regarding voluntary participation and personal confidentiality. Students who verbally agreed to participate in the survey completed the questionnaire online. The survey data were retrieved anonymously from the online survey site.

### Data analysis

Student OSCE performances were analyzed using descriptive statistics, and OSCE scores obtained for the first- and second-round tests were compared using the paired t-test. Responses to the questionnaire were analyzed using descriptive statistics. Cronbach’s alpha values were calculated to determine the internal consistency of items. Differences between responses to the survey of students with different backgrounds were analyzed using the independent t-test. Students were divided into two age groups about median age (24.0 years). Correlation coefficients were calculated to establish relationships between student perceptions of e-learning and e-learning readiness. The analysis was performed using SPSS Ver. 25.0, and statistical significance was accepted for *p* values of < 0.05.

## Results

### Participant demographics

All year-3 students participated in both OSCE rounds (*n* = 43). Sixteen (37%) were female, and 27 (63%) were male. Student ages ranged from 23 to 35 years (M = 26.56 years; SD = 3.48).

### Improvements in student performances

Student Grades Point Average (GPAs) in the third-year curriculum ranged from 2.57 to 4.33 of a possible 4.5 (M = 3.50, SD = 0.45). Student OSCE scores in the pre-test differed by age and gender (*p* < 0.01), that is, older and female students had better OSCE scores than their counterparts. However, post-test scores did not differ by gender or age. **Table 1** summarizes student performances at OSCE tests. OSCE scores improved significantly after educational intervention in all assessment domains in the OSCEs and for total scores (*p* < 0.001).

**Table 1:** Comparisons of pre- and post-intervention OSCE scores (*n* = 43).

Domains	Pre- intervention scores	Post- intervention scores	t value (*p*)
History-taking	60.78	74.41	9.10 (< .001)
Physical exams	32.88	60.55	13.16 (< .001)
Patient-physician interaction	58.02	75.53	13.86 (< .001)
Total	54.76	74.75	15.91 (< .001)

Note: Each assessment domain was awarded a maximum 100 points, and total scores were calculated using average scores.

**Fig 1:**
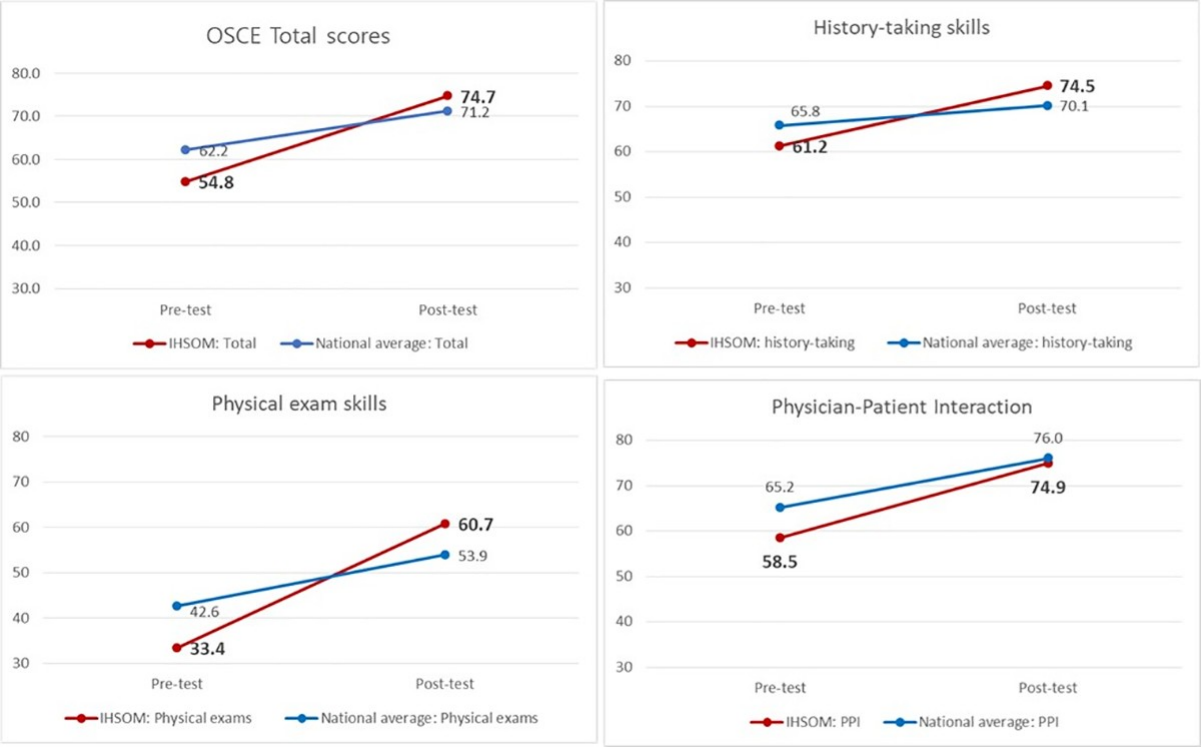
Student OSCE and national average scores pre- and post-intervention.

**Fig 1** shows comparisons of national average scores and student OSCE performances. Students’ OSCE scores in the post-test were higher than national average scores, whereas they performed lower than national average in the pre-test.

### Students’ perceived effectiveness of e-learning

Thirty-five students completed and returned the questionnaires (an 81.4% response rate). Of these respondents, 34.3% (n = 12) were female and 65.7% (n = 25) were male, and their ages ranged between 24 and 34 years (M = 26.85, SD = 3.47).

Cronbach’s alpha for items on student perceptions of e-learning was 0.90, which demonstrated a high level of international consistency of items. Table 2 provides a summary of descriptive statistics of student responses to each item. Students tended to agree with the statement that “the clinical videos aided learning clinical performance” (M = 3.66, SD = 0.76), and with the statement that “the clinical videos helped me prepare for the OSCE” (M = 3.54, SD = 0.92). Students found that the videos aided learning because “I was able to repeatedly view aspects with which I was unfamiliar” (M = 3.57, SD = 0.92). In particular, students found the videos most helpful for understanding physical examination (M = 3.63, SD = 0.88) and history-taking skills (M = 3.57, SD = 0.78, **Table 2**). Student perceptions of the effectiveness of e-learning did not differ across genders (t = .35, p = .73) nor ages (t = .52, p = .61).

**Table 2:** Descriptive statistics of student perceptions of e-learning (*n* = 35).

e-Learning resources	Mean (SD)
are helpful for learning clinical performance.	3.66 (.76)
are helpful for me to prepare for the OSCE.	3.54 (.92)
I’m generally satisfied with them.	3.09 (.92)
I’m satisfied with the quality of the resources.	2.94 (.91)
I will use them for my learning of clinical performance in the future.	3.23 (1.28)
are valuable for learning clinical performance.	3.34 (1.08)
are convenient for learning anytime any place.	3.46 (1.01)
are helpful for learning by enabling me to view repeatedly the parts that I am not familiar with.	3.57 (.92)
are useful to identify weaknesses in my clinical performance.	3.34 (.94)
Total	3.35 (.79)

1 = “Strongly disagree,” 5 = “Strongly agree”

### Student readiness for e-learning and its relationship with perceptions of e-learning

**Table 3** shows descriptive statistics of student e-learning readiness. Total scores for student e-learning readiness ranged from 33.0 to 60.0 (M = 44.40, SD = 6.16), and the mean score was 3.70 (SD = 0.51), where a score of 5 indicated highest level of readiness. Cronbach alpha for the items on student e-learning readiness was 0.85, which demonstrated an acceptable level of reliability. Student e-learning readiness was higher for males than females (*t* = 2.77, *p* < 0.05), but did not differ across ages (*t* = 1.19, *p* = 0.24).

**Table 3:** Student e-learning readiness and its associations with perceptions of e-learning (*n* = 35).

Domains	Minimum	Maximum	Mean (SD)	*r* (*p*)
Self-directed learning	2.40	5.00	3.65 (.57)	.11 (.54)
Learner control	2.00	5.00	3.55 (.70)	.25 (.14)
Motivation for learning	2.25	5.00	3.87 (.52)	.19 (.27)
Total	2.75	5.00	3.70 (.52)	.20 (.25)

1 = “Strongly disagree,” 5 = “Strongly agree”

Students’ overall perceived effectiveness of e-learning was not associated with their total e-learning readiness scores (*r* = 0.20, *p* = 0.25) nor with that of each domain of student e-learning readiness, where *p* values ranged from 0.14 to 0.54.

## Discussion

Student OSCE performances were found to improve significantly after e-learning intervention and the results obtained demonstrated e-learning effectively supports the self-directed learning of clinical skills by medical students. Our study illustrates that e-learning provides an effective means of supplementing the face-to-face teaching of clinical skills. This finding is noteworthy for the teaching and learning of clinical performance in this time when face-to-face contact in clinical settings is limited.

Despite the significant improvement in student OSCE scores after e-learning, student perceptions of its effectiveness were rather neutral. This finding may have been because the learning of clinical performance involves the use of tacit knowledge, such as diagnostic reasoning, which is a requisite competence for doctors but not easily understood by such novices as medical students [28]. Thus, it is likely that such tacit knowledge was not easy to comprehend for students by just watching a patient encounter demonstrated in the video. This finding may reflect the possible limitation of e-learning in developing clinical competency. Although e-learning is an effective and efficient approach for enhancing clinical performance at the exam setting, it needs to be supplemented with practice in real life, which involves receiving feedback on his or her clinical performance. Moreover, this finding is also likely due to student orientation to learning for assessment. Although the clinical videos presented for e-learning were not intended to teach students test-taking skills, it was our observation that students used them to prepare for the exam. Therefore, students tended to expect these video clips to show them explicitly what is assessed in the OSCE and to show them how to perform correctly in the exam rather than to gain an understanding of patient encounters and identify their learning needs.

Therefore, we make a few suggestions for improving the effectiveness of e-learning to enhance student clinical performance. First, faculty debriefing is suggested to supplement student learning from the clinical videos. During these briefings, faculty members review key points with students either online or face-to-face to help them understand the application of tacit knowledge as demonstrated by the videos. Second, clinical videos should not only demonstrate the procedures of patient encounters but also provide information on the knowledge and skills underlying them. Furthermore, future study is warranted on students’ perception of the confidence that they would have acquired in practicing with real patient after they were exposed to e-learning to investigate the impact of e-learning on enhancing clinical performance.

The student e-learning readiness scores found in our study are similar to those of Taiwanese college students reported by Hung et al. [27]. We found that students generally felt they were prepared for e-learning but that their e-learning readiness scores were not associated with their perceptions of the effectiveness of e-learning. These findings appear to reflect the backgrounds of Korean medical students, as all had experienced e-learning in high school as an important component of preparation for college entrance exams. Thus, it seems that student levels of e-learning readiness in the present study did not significantly impact their perceptions of e-learning. Therefore, our findings indicate that e-learning readiness does not significantly impact perceived learning effectiveness among those familiar with this type of learning.

We acknowledge several limitations of the present study. First, our study was conducted on a relatively small number of students recruited at a single institution. Although the response rate of our survey study was relatively high (81.4%), it can still raise the issue of non-respondent bias due to the small sample size. We made efforts to increase the response rate by sending the study participants the e-mail several times. Still, the online method of survey in our study may have had an impact on the response rate, which tends to be lower in online surveys than that of the paper-based format [29, 30]. A common way to evaluate non-response bias is to compare demographic information between respondents and potential respondents [29]. Our study showed that our survey respondent characteristics (i.e., gender and age) did not significantly differ from those of the study cohort and that nor did their perceptions of the effectiveness of e-learning differ across respondent characteristics. Still, our study does not provide clear evidence on whether the nonrespondents may have had responded differently from the respondents. Therefore, a larger-scale study is warranted to enhance the generalizability of our findings. Second, the participants in the study were those who have been exposed to high use of technologies and had already been experienced with e-learning. Thus, we did not measure student efficacy on the computer / Internet self-efficacy in our study participants, which were included in the Hung’s [27] original scale on the learner readiness for e-learning. Hence, our findings may not be generalizable to students with limited experience of e-learning or computer usage. A larger-scale study is warranted to investigate the effectiveness or impacts of e-learning in students with different levels of exposure to e-learning and computer usage.

In fact, this study has been planned and conducted prior to the COVID-19 pandemic. Later, while we organized our findings, pandemic began, and we thought the findings could help solve our struggles with medical education while keeping social distancing. In fact, as a result of teaching e-learning to students using the same program after pandemic, we were able to confirm similar learning effects continuously.

In conclusion, this study shows student OSCE performance improved significantly after an educational intervention using e-learning, which indicate its effectiveness to support student learning of clinical performance. Students’ perceived effectiveness of e-learning appears not to be affected by their preparedness for such a learning environment. Still, student perceptions of the effectiveness of e-learning were rather neutral. Implications for the design of e-learning resources and faculty use of such resources are suggested for more effective use of e-learning to enhance medical student clinical performances.

## Supporting information

S1 File (XLS)

## List of abbreviations

OSCE: Objective Structured Clinical Examination
GPA: Grade Point Average

## References

[1] Woolliscroft JO. Innovation in response to the COVID-19 pandemic crisis. Acad Med. 2020;95(8):1140–2. doi: 10.1097/ACM.0000000000003402 00001888-900000000-97219. 32282372 PMC7188042

[2] Rose S. Medical student education in the time of COVID-19. JAMA. 2020; 323(21):2131–2. doi: 10.1001/jama.2020.5227 32232420

[3] Klamen DL, Williams R, Hingle S. Getting real: aligning the learning needs of clerkship students with the current clinical environment. Acad Med. 2019;94(1):53–8. doi: 10.1097/ACM.0000000000002434 . 30157091

[4] Lee Klamen D. Getting real: embracing the conditions of the third-year clerkship and reimagining the curriculum to enable deliberate practice. Acad Med. 2015;90(10):1314–7. doi: 10.1097/ACM.0000000000000733 . 25901873

[5] Roh H. Educational strategies for clinical and technical skills performance. Korean Med Educ Rev. 2016;18(3):132–44. KJD:ART002160522.

[6] Kurtz S, Silverman J, Draper J. Teaching and learning communication skills in medicine. 2nd ed ed. Oxford: Radcliffe Medical; 2004.

[7] Han H, Roberts NK, Korte R. Learning in the real place: medical students’ learning and socialization in clerkships at one medical school. Acad Med. 2015;90(2):231–9. doi: 10.1097/ACM.0000000000000544 . 25354072

[8] Young I, Montgomery K, Kearns P, Hayward S, Mellanby E. The benefits of a peer-assisted mock OSCE. Clin Teach. 2014;11(3):214–8. doi: 10.1111/tct.12112 . 24802924

[9] Hopwood J, Myers G, Sturrock A. Twelve tips for conducting a virtual OSCE. Med Teach. 2020:1–4. Epub 2020/10/20. doi: 10.1080/0142159X.2020.1830961 . 33078984

[10] Gordon M, Patricio M, Horne L, Muston A, Alston SR, Pammi M, et al. Developments in medical education in response to the COVID-19 pandemic: a rapid BEME systematic review: BEME Guide No. 63. Med Teach. 2020;42(11):1202–15. Epub 2020/08/26. doi: 10.1080/0142159X.2020.1807484 . 32847456

[11] Ertmer PA, Newby TJ. The expert learner: strategic, self-regulated, and reflective. Instr Sci. 1996;24(1):1–24. doi: 10.1007/bf00156001 WOS:A1996UH73300002.

[12] Schön DA. The reflective practitioner: how professionals think in action. New York: Basic Books; 1983.

[13] Tagawa M, Imanaka H. Reflection and self-directed and group learning improve OSCE scores. Clin Teach. 2010;7(4):266–70. doi: 10.1111/j.1743-498X.2010.00377.x . 21134204

[14] White CB, Ross PT, Gruppen LD. Remediating students’ failed OSCE performances at one school: the effects of self-assessment, reflection, and feedback. Acad Med. 2009;84(5):651–4. doi: 10.1097/ACM.0b013e31819fb9de WOS:000267655300021. 19704203

[15] Bonk CJ, Lee MM, Kou XJ, Xu SY, Sheu FR. Understanding the self-directed online learning preferences, goals, achievements, and challenges of MIT OpenCourseWare subscribers. Educa Technol Soc. 2015;18(2):349–65. WOS:000354884000026.

[16] Scott K, Morris A, Marais B. Medical student use of digital learning resources. Clin Teach. 2018;15(1):29–33. Epub 2017/03/16. doi: 10.1111/tct.12630 28300343. 28300343

[17] Frehywot S, Vovides Y, Talib Z, Mikhail N, Ross H, Wohltjen H, et al. E-learning in medical education in resource constrained low- and middle-income countries. Hum Resour Health. 2013;11(4). doi: 10.1186/1478-4491-11-4 ; PubMed Central PMCID: PMC3584907. 23379467 PMC3584907

[18] Kim KJ, Kim G. Development of e-learning in medical education: 10 years’ experience of Korean medical schools. Korean J Med Educ. 2019;31(3):205–14. Epub 2019/08/26. doi: 10.3946/kjme.2019.131 ; PubMed Central PMCID: PMC6715898. 31455050 PMC6715898

[19] Moore JL, Dickson-Deane C, Galyen K. e-Learning, online learning, and distance learning environments: are they the same? Internet High Educ. 2011;14(2):129–35. doi: 10.1016/j.iheduc.2010.10.001 WOS:000288887800009.

[20] Pei L, Wu H. Does online learning work better than offline learning in undergraduate medical education? A systematic review and meta-analysis. Med Educ Online. 2019;24(1):1666538. doi: 10.1080/10872981.2019.1666538 ; PubMed Central PMCID: PMC6758693. 31526248 PMC6758693

[21] Al-Shorbaji N, Atun R, Car J, Majeed A, Erica W. eLearning for undergraduate health professional education. World Health Organization, 2015.

[22] Jang HW, Kim K-J. Use of online clinical videos for clinical skills training for medical students: benefits and challenges. BMC Med Educ. 2014;14. doi: 10.1186/1472-6920-14-14 WOS:000334463500001. 24650290 PMC3994418

[23] Dinscore A, Andres A. Surgical videos online: a survey of prominent sources and future trends. Med Ref Serv Q. 2010;29(1):10–27. doi: 10.1080/02763860903484996 . 20391161

[24] Cook DA. The failure of e-learning research to inform educational practice, and what we can do about it. Med Teach. 2009;31(2):158–62. doi: 10.1080/01421590802691393 19330674

[25] Lu HP, Chiou MJ. The impact of individual differences on e-learning system satisfaction: a contingency approach. Br J of Educ Technol. 2010;41(2):307–23. doi: 10.1111/j.1467-8535.2009.00937.x WOS:000274450300031.

[26] Parkes M, Stein S, Reading C. Student preparedness for university e-learning environments. Internet High Educ. 2015;25:1–10. doi: 10.1016/j.iheduc.2014.10.002 WOS:000351980800001.

[27] Hung M-L, Chou C, Chen C-H, Own Z-Y. Learner readiness for online learning: scale development and student perceptions. Comput Educ. 2010;55(3):1080–90. doi: 10.1016/j.compedu.2010.05.004 WOS:000280985600016.

[28] Heiberg Engel PJ. Tacit knowledge and visual expertise in medical diagnostic reasoning: implications for medical education. Med Teach. 2008;30(7):e184–8. doi: 10.1080/01421590802144260 . 18777417

[29] Phillips AW, Reddy S, Durning SJ. Improving response rates and evaluating nonresponse bias in surveys: AMEE guide no. 102. Med Teach. 2016;38(3):217–28. doi: 10.3109/0142159X.2015.1105945 . 26648511

[30] Ebert JF, Huibers L, Christensen B, Christensen MB. Paper- or Web-based questionnaire invitations as a method for data collection: cross-sectional comparative study of differences in response rate, completeness of data, and financial cost. J Med Internet Res. 2018; 20(1): e24. doi: 10.2196/jmir.8353 29362206 PMC5801515

